# A Statistical Report of Diseases of the Ear

**Published:** 1870-09

**Authors:** E. L. Holmes

**Affiliations:** Professor of Ophthalmology and Otology, Rush Medical College, Surgeon to Chicago Charitable Eye and Ear Infirmary


ARTICLE II.—*A Statistical Report of Diseases of the Ear.* By E. L. HOLMES, M.D., Professor of Ophthalmology and Otology, Rush Medical College, Surgeon to Chicago Charitable Eye and Ear Infirmary.

(Read before the State Medical Society, at Dixon, May, 1870.)

The treatment of diseases of the ear, as also of the eye, has fallen, almost entirely, into the hands of specialists, wherever patients can obtain their services.

The chief cause of this is undoubtedly found in the fact that physicians seldom become interested either in the study or treatment of these diseases.

So far is this true, that scarcely a single man of eminence, as far as I know, in Europe or America, engaged in general medical and surgical practice, is willing to express a decided opinion in severe or obscure cases of diseases of the eye or ear.

Ophthalmology and Otology have, therefore, almost from necessity, become emphatically special departments of medicine and surgery.

I have, at previous meetings of this society, expressed my opinions as to what extent the oculist should be a thoroughly educated physician; and to what extent general practitioners, not residing in large towns, should comprehend the theory and practice of ophthalmic medicine.

What I have said regarding ophthalmology may be said, I believe, regarding aural science. The physician upon whom alone a community can depend for aid and council in disease, should be able to give relief in a large proportion of acute cases of aural disease.

Although certain classes of aural diseases are exceedingly obscure, there are many others which require comparatively little study and clinical experience to enable the practitioner to render inestimable service to his patients. There is no excuse for ignorance of this class of cases, in the general practitioner.

As compared with the literature of ophthalmology, that of otology is exceedingly limited. Hence there is less excuse, if possible, for the practitioner in neglecting the simple and practical portions of this department.

The number of persons in every community affected with diseases of the ear is by no means small. I think it is well established, that of the aggregate number of patients requiring medical aid, about one-twelfth suffer from some disease of the eye. The reports of different infirmaries present a variable proportion of cases of ear diseases. I think, however, that the average proportion of patients with aural diseases is about one-fifth of the number of patients with diseases of the eye.

In my own experience during fourteen years in private and hospital practice, the aggregate number of eye cases has been 8,982; of ear cases, 1,735. Of this number 1,076 were treated in private and 659 in dispensary practice. I have observed that relatively a less number of indigent patients were affected with diseases of the ear than of the eye.

In the following table the cases are arranged in accordance with the diagnosis, recorded in my daily journal. The modern system of classification is somewhat different from that adopted by some authors a few years ago. In a very large number of instances the cases were recorded without true scientific classification, as, for example, in cases of very long duration, where the chief objective symptoms were found to be otorrhœa and perforated membrana tympani, with more or less excoriation of the external meatus; for example, also, cases of very long duration in which there was found to be simply perforation without otorrhœa.

I have never observed a single case in which I was confident symptoms of inflammation were confined to the membrana tympani alone, independent of disease of the middle ear or external meatus.

TABLE OF DISEASES OF THE EAR.

| | |
|---|---|
| Perforation of Memb. Tympani—Otorrhœa, | 321 |
| " " " without Otorrhœa, | 17 |
| Polypus Auris, | 27 |
| Chronic Inflammation—External Meatus, | 71 |
| Acute Inflammation, External Meatus and Memb. Tympani, | 42 |
| Furuncle of External Meatus, | 31 |
| Caries of External Meatus, | 2 |
| Accumulation of Cerumen, | 143 |
| Exostosis of External Meatus, | 2 |
| Foreign Bodies in External Meatus, | 17 |
| Burn of Auricle and External Meatus, (lime, melted iron, etc.) | 8 |
| Eczema of Auricle and External Meatus, | 31 |
| Tumor of Auricle, | 4 |
| Frost Bite of Auricle, | 3 |
| Injury of Auricle, | 7 |
| Ecchymosis of Auricle, | 3 |
| Congenital Deficiency of Auricle and External Meatus, | 3 |
| Mastoid Abscess, | 4 |
| Caries of Mastoid Process, | 2 |
| Maniere's Disease (?), | 2 |
| Chronic Inflammation, Middle Ear, | 796 |
| Acute " " " | 57 |
| " " " " and Labyrinth—death, | 2 |
| Abnormal Acuteness of Hearing, | 1 |
| Tinnitus, (no apparent cause), | 3 |
| Deaf Mutes, | 38 |
| Total Deafness, (Disease of Labyrinth—Auditory Nerve,) | 23 |
| Deafness, (Syphilitic Disease,) | 3 |
| Calcareous Deposits in Memb. Tympani, | 3 |
| Unclassified, | 68 |
| Total, | 1,735 |

